# Methods for storage and determination of tryptophan and serotonin in bovine blood by high-performance liquid chromatography with fluorescence detection

**DOI:** 10.1016/j.vas.2026.100849

**Published:** 2026-09-01

**Authors:** Ryana Cristina Markmann, Maximiliane Alavarse Zambom, Gilberto Costa Braga, Julio César Meyer, Maria Luiza Fischer, Sidnei Antônio Lopes, David Lee Harmon, Ériton Egidio Lisboa Valente

**Affiliations:** a Department of Animal Science, Western Paraná State University, Marechal Cândido Rondon, PR, 85960-128, Brazil; b Department of Agronomy, Western Paraná State University, Marechal Cândido Rondon, PR, 85960-128, Brazil; c Department of Animal Science, Universidade Federal de Viçosa, Viçosa, MG, 36570-900, Brazil; d Department of Animal and Food Science, University of Kentucky, Lexington, KY, 40506, USA

**Keywords:** Blood fraction, HPLC, Method validation, Neurotransmitter, Sample preservation

## Abstract

Tryptophan (TRP) and serotonin (5-HT) play important biological roles, including neurotransmission. However, the preservation and quantification of TRP and 5-HT in blood samples are challenging due to their instability. In addition, methods for their determination in biological samples using high-performance liquid chromatography with fluorescence detection (HPLC-FLD) remain limited. This study aimed to develop methods for the preservation and quantification of 5-HT and TRP in blood using HPLC-FLD. Mobile phases with different pH values (3.5, 4.0, and 4.5) and methanol-to-buffer ratios (25:75, 20:80, and 15:85) were evaluated. A stability study using different preservatives (perchloric acid, oxalic acid, oxalic acid + glycine, EDTA, EDTA + perchloric acid, Na_2_S_2_O_5_, Na_2_S_2_O_5_ + EDTA, 15% methanol, and 30% methanol) and storage temperatures was conducted. TRP and 5-HT concentrations were determined in whole blood, serum, heparinized plasma, and EDTA plasma. The optimized mobile phase consisted of methanol and KH_2_PO_4_ buffer (pH 3.5) at a 20:80 ratio. Intraday and interday precision were 2.20% and 4.43% for TRP, and 2.36% and 4.96% for 5-HT, respectively. Mean accuracy was 105.2% for TRP and 99.4% for 5-HT. Serum samples preserved with 30% methanol showed no significant changes (P > 0.05) in TRP and 5-HT concentrations when stored at −20°C or −80°C. TRP exhibited minor variation among blood fractions, whereas 5-HT was higher in serum. The proposed analytical method is sensitive and reliable for quantifying TRP and 5-HT in blood. Methanol (30%) effectively preserved TRP and 5-HT during frozen storage for at least 56 days.

## Highlights

• Preservation and quantification of Tryptophan and serotonin samples are challenging.
• Tryptophan and serotonin are unstable, even when stored at −80°C.
• A sensitive HPLC-FLD method was developed for serotonin quantification.
• Methanol effectively preserves tryptophan and serotonin in blood samples.

## Graphical abstract

**Figure:**
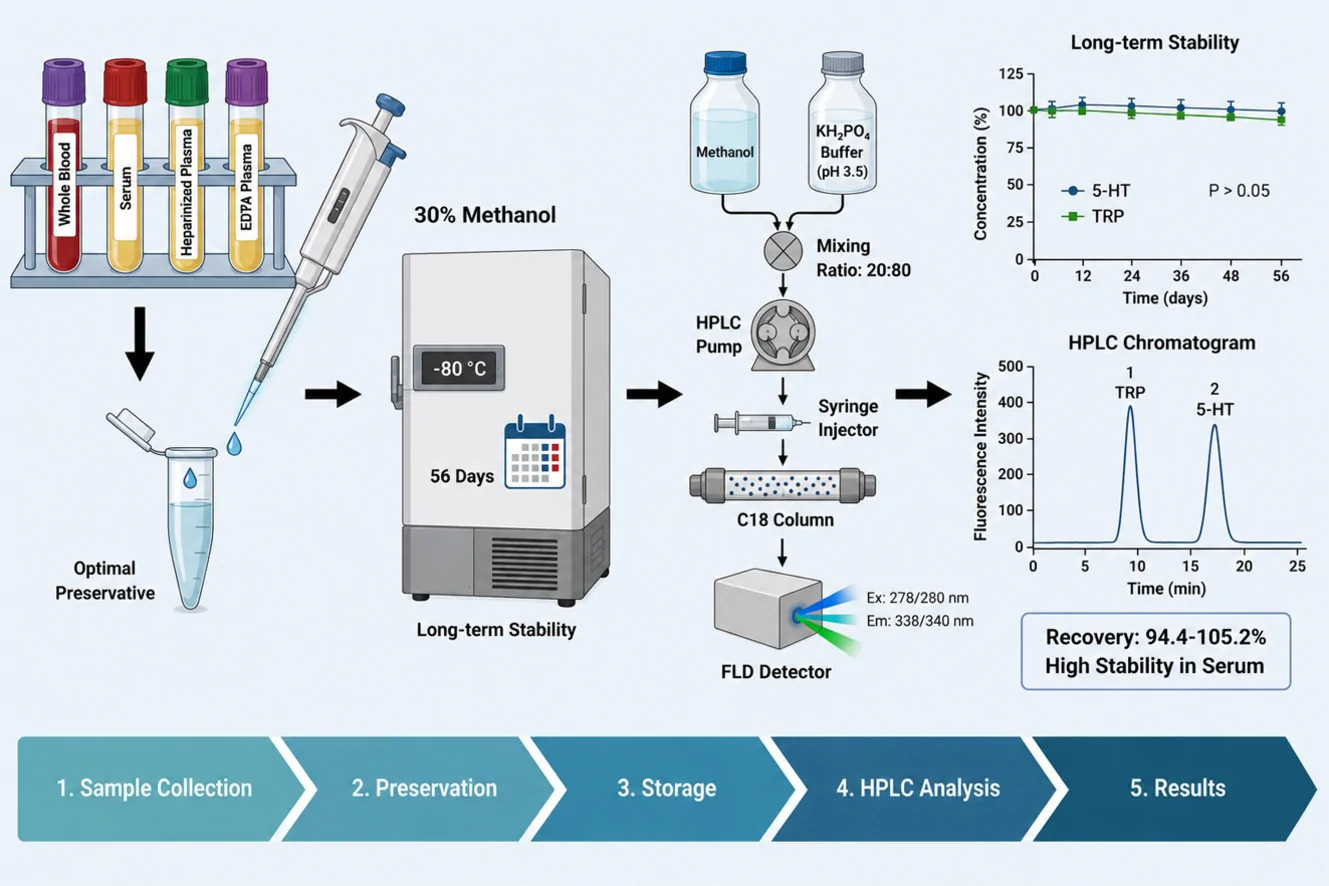

## Introduction

Tryptophan (TRP) and serotonin (5-HT) have essential roles in living beings (Yao et al., 2011). The neurotransmitter 5-HT is a biogenic monoamine synthesized from the essential amino acid TRP (Keszthelyi et al., 2009). Serotonin acts on peripheral tissues and on the central nervous system as a neurotransmitter involved in the regulation of a broad range of physiological and behavioral processes, including reproductive function, stress responses, feeding behavior, circadian and activity cycles, locomotor activity, immune activity, and inflammatory processes (Bacqué-Cazenave et al., 2020; Berger et al., 2009).

The determination of TRP and 5-HT is often performed by chromatographic methods using sophisticated equipment due to their low concentrations in blood, frequently involving derivatization, electrochemical detection, or mass spectrometry (Christofides et al., 2006; Fujino et al., 2003; Hubbard et al., 2010; Sakaguchi et al., 2015). Although alternative chromatographic methods using fluorescent detectors are more economical and can provide excellent results (Sa et al., 2012). There is scarce information about HPLC-FLD methods for evaluating different blood fractions.

The quantification of 5-HT is also challenging due to its instability, as it is susceptible to biochemical and chemical oxidation reactions (Szeitz and Bandiera, 2018), and sensitive to light exposure and to environmental variations such as pH and temperature (Corona-Avendaño et al., 2005; Sánchez-Rivera et al., 2003). Several preservation and analytical methods have been developed to prevent compound degradation; however, each sample type requires specific procedures.

Platelets store most of the circulating 5-HT, which is released during the coagulation process (Bertrand and Bertrand, 2010; Szeitz and Bandiera, 2018). Therefore, clot formation and the procedure used for the separation of blood fractions can markedly affect analytical results. The objective of this study is to develop a rapid and accurate method for storage and simultaneous quantification of TRP and 5-HT using high-performance liquid chromatography with fluorescence detection (HPLC-FLD) in different blood fractions.

## Material and methods

The experimental procedures were approved by the Ethics Committee on the Use of Animals of Western Parana State University under protocol 018/2025.

### Chemicals and solutions

Perchloric acid (PA) was supplied by Anidrol (São Paulo, Brazil). Methanol (HPLC grade), serotonin hydrochloride (98%), and L-tryptophan (99%) were supplied by Sigma–Aldrich (São Paulo, Brazil).

Stock solutions were prepared at concentrations ranging from 50 to 75 µM using ultrapure water. After the appropriate dilution, the working solutions were filtered through a 0.45 µm cellulose membrane in syringe filters and transferred into vials.

### Optimizing instruments and chromatography conditions

Analyses were conducted using high-performance liquid chromatography (HPLC; model 2695; Waters, Milford, MA, USA) equipped with a fluorescence detector (model 2475; Waters, Milford, MA, USA) and a Shim-pack GIST PFPP column (3 μm, 2.1 × 150 mm; Shimadzu, Kyoto, Japan).

Chromatographic separation was performed using a mobile phase composed of 50 mM potassium phosphate buffer and methanol. To determine the optimal mobile phase composition, different hydrogen ion potentials (pH 3.5, 4.0, and 4.5) and methanol:buffer ratios (25:75, 20:80, and 15:85, v/v) were evaluated. The mobile phase was filtered through a 0.45 µm membrane and degassed for 20 min using an ultrasonic bath.

The fluorescence detector was operated with excitation and emission wavelengths set at 278 and 338 nm, respectively. The mobile phase had a flow rate of 1.0 mL/ min under isocratic conditions. The oven was set to 35 °C. A volume of 20 μL of each prepared sample was injected into the HPLC system. A single needle wash was performed for 13 s using ultrapure water containing 30% methanol. The sample loop volume was set at 50 µL, and the total run time was 7 min.

### Chromatographic method validation

Method validation was conducted in compliance with the Harmonization of Technical Requirements for Pharmaceuticals for Human Use M10 guidelines (ICH, 2022). Validation parameters included linearity, lower limits of detection (LLOD) and quantification (LLOQ), carry over, reinjection reproducibility, short-term stability, three freeze–thaw cycles, precision, and accuracy.

Peak resolution (Rs) was calculated as the following equation:

$$\text{Rs}=\frac{2\left(t_{R2}-\;t_{R2}\right)}{W_{b1}+\;W_{b2}}$$

Where t_R1_ and t_R2_ are retention times of the two peaks (measured from injection to peak apex), and W_b1_ and W_b2_ are peak widths at the baseline, calculated by extrapolating the straight sides of the peak to the baseline.

Linearity was evaluated using standard solutions at eight concentrations, analyzed in triplicate over three different days. For TRP, the concentrations were 1500, 750, 375, 187.5, 93.8, 46.9, 23.4, and 11.7 nM. For 5-HT, the evaluated concentrations were 1622, 811, 405.5, 202.8, 101.4, 50.7, 25.3, and 12.7 nM. Calibration curves were constructed by plotting peak areas against standard concentrations, and linearity was assessed using linear regression analysis.

The LLOD was established as the lowest concentration in the standard solution that produced a signal-to-noise (S/N) ratio of 3. The LLOQ was established as the lowest concentration that produced a signal-to-noise ratio of 10.

Carry-over was assessed by analyzing blank samples in triplicate immediately after injection of the calibration standard at the highest standard concentration.

Reinjection reproducibility was determined by reinjecting a sequence comprising calibration standards and 5 replicates of low-, medium-, and high-standard concentrations (16, 160, and 1600 nM).

For short-term stability evaluation, samples spiked at 160 nM and maintained at room temperature (23.5 ± 0.68°C) for 0, 6, 12, 18, and 24 h during HPLC analysis were analyzed in triplicate. To evaluate storage losses, three freeze–thaw cycles were performed in triplicate, with samples stored frozen for at least 12 hours between cycles and thawed at room temperature for 1 h prior to analysis.

Precision was determined by analyzing 3 replicates per run at each QC concentration level (LLOQ, low, medium, high, upper limit of quantification) across 6 runs over 3 days. Precision was expressed as the coefficient of variation (CV).

Method accuracy was evaluated by recovery experiments. Serum samples were spiked with standard solutions of TRP and 5-HT at five concentrations corresponding to 1600, 800, 400, 200 and 100 nM with 3 replicates.

### Sample collection

For method validation and storage stability evaluation, blood samples were collected from six lactating Holstein cows (648.9 ± 26.5 kg) using vacuum tubes with clot activator. Tubes were kept in the dark at room temperature for 40 min to allow clot formation, then centrifuged at 2,000 × g for 30 min.

An independent study was conducted to evaluate 5-HT and TRP concentrations in different blood fractions. For this experiment, six 14-month-old Holstein bulls (436.8 ± 36.4 kg) were used. The physiological differences between the animals used in the two experiments were not expected to affect the objective of the second experiment, because comparisons among blood fractions were performed within the same animals. Blood samples from each bull were collected into three different vacuum tubes containing specific additives to obtain the respective blood fractions. Clot activator tubes were used for serum separation, EDTA_2_Na tubes for plasma collection, and heparinized tubes for plasma and whole-blood analyses. After collecting, tubes containing the clot activator were protected from light and maintained at room temperature for 40 min to allow clot formation before centrifugation. An aliquot from the heparin tube was reserved for whole-blood analyses and was not immediately centrifuged. The remaining tubes were centrifuged immediately after collection at 3,500 rpm for 15 min. Aliquots of blood fractions were transferred to 2-mL tubes, and 30% of methanol was added. Samples were stored at -80 °C until analysis (30 d).

### Optimizing serum preparation

Blood samples were analyzed as described by (Valente et al., 2024) with modifications. In a 2-mL microcentrifuge tube, an aliquot of 250 µL of serum and 50 µL of 4 M HClO₄ were combined. Samples were vortexed and centrifuged at 14,000 × g for 10 min. Subsequently, 35 µL of 4 M KOH was added to maintain the sample pH above 2, followed by centrifugation at 14,000 × g for 5 min. An aliquot of the supernatant was diluted 1/10 with ultrapure water, filtered through a 0.45 µm cellulose membrane and transferred into vials to be subsequently analyzed by HPLC.

### Optimizing storage conditions and stability of blood samples

Storage stability was evaluated using blood samples collected from six Holstein lactating cows (648.9 ± 26.5 kg) using vacuum tubes containing clot activator. Samples were protected from light and maintained at room temperature for 40 min to allow clot formation, followed by centrifugation for 15 min at 3,500 rpm.

The treatments consisted of the addition of the following preservative compounds: perchloric acid (0.67 M); oxalic acid (0.01 M); oxalic acid (0.01 M) + glycine (0.17 M); disodium ethylenediaminetetraacetic acid (EDTA, 0.17 M); EDTA (0.17 M) + perchloric acid (0.67 M); sodium metabisulfite (0.08 M); EDTA (0.17 M) + sodium metabisulfite (0.08 M); methanol 15%; methanol 30%. For the control, no preservative compounds were added.

Aliquots of 250 µL of serum were transferred into 2 mL and 50 μL of each preservative solution were added to the treatments, except for the 30% methanol treatment, in which 100 μL was added. For the controls, 250 μL of serum was used alone. Each treatment was performed with six replicates. The microcentrifuge tubes were properly identified and stored at 4°C. Serum TRP and 5-HT concentrations were analyzed at three time points: immediately prior to storage, after 2 days of storage, and after 4 days of storage.

The serums with the best short-term stability (storage at 4°C for 4 days) were selected for long-term stability evaluation. A preservative-free serum was also included in the study. These samples were stored at −20°C and −80°C for up to 56 days. Accordingly, the evaluated treatments were serum (control), serum with HClO₄ and EDTA, and serum with HClO₄ and 30% methanol. TRP and 5-HT concentrations were determined in freshly prepared samples and in samples stored for 7, 14, 28, and 56 days. Samples were aliquoted immediately after preparation, and each aliquot was thawed only once, immediately before analysis at the designated storage time, thereby avoiding repeated freeze–thaw cycles.

### Statistical analysis

All data were analyzed as a completely randomized design using the MIXED procedure of SAS (OnDemand for Academics; SAS Institute Inc., Cary, NC, USA). For the stability evaluations, the following model was applied:

$$Y_{\text{ij}}=\mu +\text{TR}T_{i}+\;\text{Tim}e_{j}+\text{TRTxTim}e_{\text{ij}}+e_{\text{ij}}$$

where *Yᵢⱼ* is the dependent variable, *μ* is the overall mean, *TRTᵢ* is the fixed effect of treatment, *Timeⱼ* is the fixed effect of storage time, *TRT × Timeᵢⱼ* is the interaction between fixed effects, and *eᵢⱼ* is the random error.

Data from blood fractions were analyzed according to the following model:

$$Y_{\text{ijkl}}=\mu +F_{i}+\;\text{Tim}e_{j}+\;A_{k}+\;P_{l}+\text{FxTim}e_{\text{ij}}+e_{\text{ijkl}}$$

where *Yᵢⱼₖₗ* is the dependent variable, *μ* is the overall mean, *Fᵢ* is the fixed effect of blood fraction, *Timeⱼ* is the fixed effect of sampling time, *Aₖ* is the random effect of animal, *Pₗ* is the random effect of period, *F × Timeᵢⱼ* is the interaction between fixed effects, and *eᵢⱼₖₗ* is the random error.

Normality of residuals was evaluated using the Shapiro–Wilk test (Shapiro and Wilk, 1965) with the UNIVARIATE procedure of SAS. Data for TRP and 5-HT were transformed using the Box–Cox transformation (Box and Cox, 1964) due to deviations from normality, as described by:

$$Y\left(\lambda \right)=\left\{ \begin{array}{c} \frac{Y^{\lambda }-1}{\lambda },\;if\;\lambda \;\neq \;0\\ \log y,\;if\;\lambda =\;0 \end{array}\right.$$

where the lambda (λ) value for each variable was estimated using the TRANSREG procedure of SAS with the Box–Cox model, with λ ranging from −2 to 2.

After statistical analysis, data were back-transformed to concentration units for presentation purposes. The standard error (SEM) of the back-transformed data was calculated from the confidence limits of the transformed data as follows:

$$\text{SEM}=\frac{\;\text{upper}\;\text{CL}\;-\text{lower}\;\text{CL}}{3.92}$$

Where upper CL is back-transformed upper confidence limit and lower CL is back-transformed lower confidence limit.

Pairwise comparisons were performed using the least significant difference (LSD) test. Statistical significance was declared at *P* ≤ 0.05, and trends were discussed when 0.05 < *P* ≤ 0.10.

## Results and discussion

We report a storage sample method to prevent degradation and a sensitive isocratic HPLC method with fluorescent detection for the simultaneous determination of TRP and 5-HT in blood. The chromatographic conditions of the method were suitable for analysis in different blood fractions.

### Chromatographic method

The reduction of pH from 4.5 to 3.5 increased the retention time of TRP by 4.75% and reduced the peak area by 8.7%. However, it caused a 30.8% reduction in the retention time of 5-HT and a 1.9% increase in peak area (Table 1). Resolution remained above 2 for all pH values. Reverse phase pentafluorophenyl columns can operate under low pH conditions (typically pH 2–3) and exhibit strong affinity for amine groups, as reported by manufacturers. These columns have been successfully used to determine neurotransmitters (Suominen et al., 2013; Valente et al., 2024). Reducing the pH from 4.5 to 3.5 resulted in a significant increase in the 5-HT peak area and a reduction in retention time, with only a slight decrease in TRP peak area. Because serum concentrations of 5-HT are several-fold lower than those of TRP (Valente et al., 2021), optimization of the chromatographic method was prioritized for this neurotransmitter.

**Table 1:** Mobile phase pH on the retention time, peak area, and chromatographic resolution of tryptophan (TRP) and serotonin (5-HT).

Item	pH^1^		
	3.5	4	4.5
TRP			
Retention time, min	4.40 ± 0.001	4.27 ± 0.001	4.20 ± 0.008
Area	3898 ± 59	4022 ± 49	4268 ± 36
5-HT			
Retention time, min	5.74 ± 0.049	6.95 ± 0.019	8.29 ± 0.245
Area	15516 ± 59	14981 ± 147	15233 ± 60
Peak resolution	2.59 ± 0.03	4.68 ±0.04	6.07 ± 0.04

1 methanol to phosphate buffer ratio of 20:80. Average ± standard error of mean (n=4).

Increasing the proportion of methanol in the mobile phase gradually reduced the retention time and increased the peak area of TRP and 5-HT (Table 2). However, increasing methanol to 25% reduced peak resolution to below 2 and increased system pressure to near the column limit (4000 psi). A methanol proportion of 20% was considered optimal, as higher proportions increased column backpressure to near its operational limit, potentially compromising column lifespan. Furthermore, 20% methanol represented the breakpoint at which the peak resolution reached a value of 2, indicating adequate chromatographic separation between adjacent peaks (Epshtein, 2020).

**Table 2:** Proportion of methanol (MeOH) and phosphate buffer Mobile phase (KH_2_PO_4_) on the retention time, peak area, and chromatographic resolution of tryptophan (TRP) and serotonin (5-HT).

Item	MeOH:KH_2_PO_4_		
	15:85	20:80	25:75
TRP			
Retention time, min	4.97 ± 0.003	4.40 ± 0.001	3.94 ± 0.002
Area	3803 ± 12	3898 ± 59	3930 ± 14
5-HT			
Retention time, min	6.65 ± 0.005	5.74 ± 0.049	4.95 ± 0.001
Area	14469 ± 62	15516 ± 59	16382 ± 84
Peak resolution	2.33 ± 0.03	2.59 ± 0.03	1.90 ± 0.02

^1^Mobile phase pH of 3.5. Average ± standard error of mean (n=4).

**Fig. 1:**
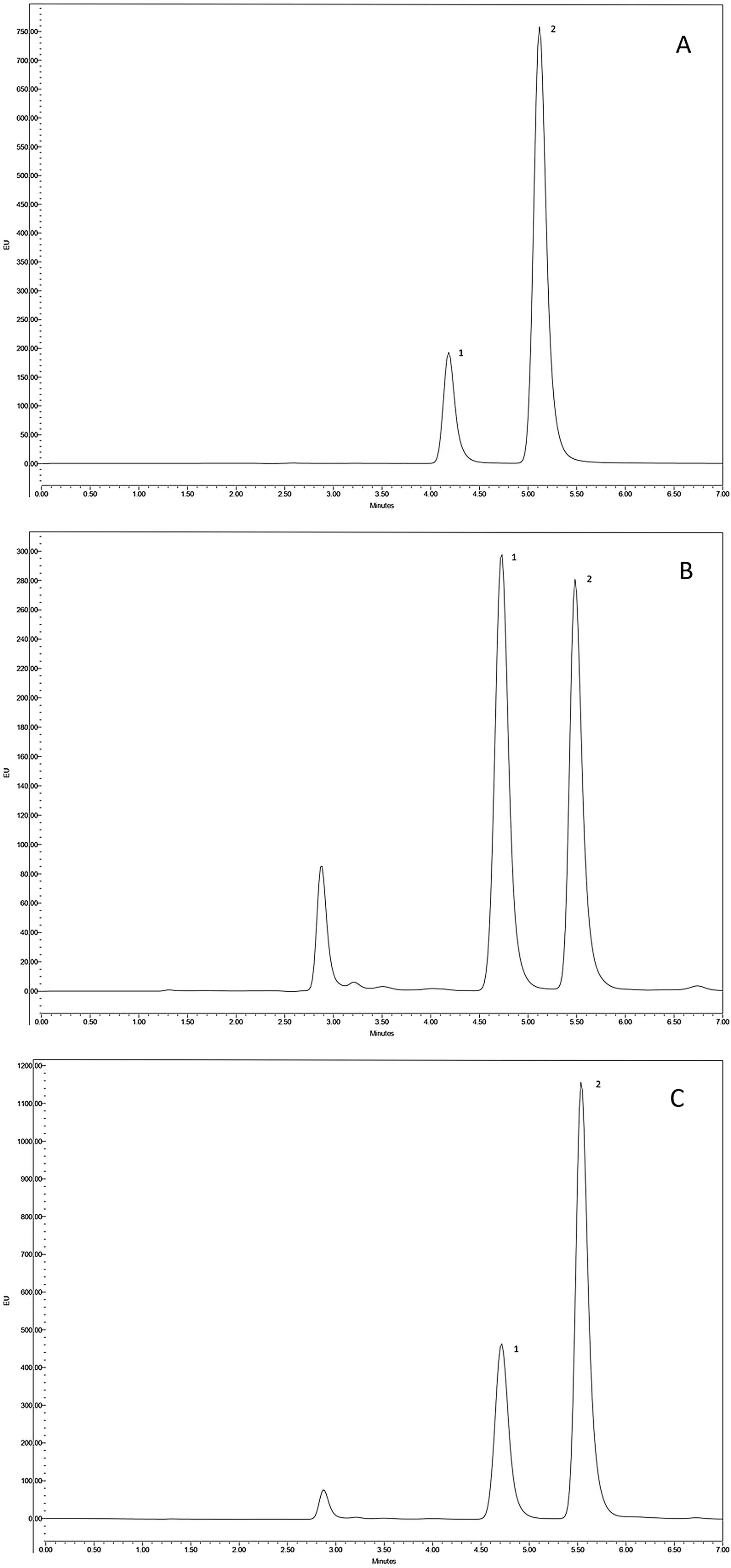
Chromatogram using mobile phase with 20:80 ratio of methanol and KH_2_PO_4_ with pH 3.5, showing tryptophan (TRP, 1) and serotonin (5-HT, 2) peaks. (A) Mixture of standards; (B) blood serum; (C) blood serum spiked with TRP and 5-HT.

The mobile phase consisting of 20% methanol and 80% KH₂PO₄ at pH 3.5 under isocratic conditions was the condition that combined the shortest run times with the highest peak areas and resolution without exceeding the column pressure limit (Fig. 1A).

### Chromatographic method validation

The method had excellent linearity over a wide concentration range for TRP (11.7 to 1500 nM) and 5-HT (12.7 to 1622 nM). The calibration curves showed correlation coefficients of 0.999 for both TRP and 5-HT. Method linearity was confirmed by a direct proportional relationship between chromatographic peak area and analyte concentration over a wide concentration range. In addition, the calibration curves showed high correlation coefficients for both TRP and 5-HT, indicating high method reliability.

The average CV for reinjection reproducibility for the TRP was 1.58% with values of 1.86%, 1.66% and 1.22% at concentrations of 1667 nM, 167 nM and 16.7 nM, respectively. For 5-HT, the average CV for reinjection reproducibility was 1.75%, with values of 2.45%, 1.78%, and 1.01% at concentrations of 1833, 183, and 18.3 nM, respectively. The average CV values below 2% for both TRP and 5-HT indicate that the method showed excellent reproducibility.

The LLOD and LLOQ values obtained for TRP were 2.58 and 8.60 nM, respectively. For 5-HT, the LLOD was 0.78 nM and the LLOQ was 2.60 nM. The LLOQ values obtained were lower than those reported in the literature for TRP (Islam et al., 2016; Sa et al., 2012; Vignau et al., 2004) and 5-HT (Bearcroft et al., 1995; De Benedetto et al., 2014; Islam et al., 2016) using HPLC with a fluorescent detector. The LLOD and LLOQ further demonstrate the high sensitivity of the method, enabling the detection of low concentrations of TRP and 5-HT.

The intraday and interday CV were 2.20% and 4.43% for TRP, and 2.36% and 4.96% for 5-HT, respectively (Table 3). Precision was evaluated in terms of repeatability, with the CV being the most common statistical parameter. As a general guideline, CV values below 5% are considered acceptable for most quantitative analytical methods. Intraday precision was assessed by the dispersion of results obtained under identical operating conditions over a short time interval, whereas interday precision is more susceptible to external sources of variability. Intraday CV values were excellent, with values close to 2% for both TRP and 5-HT. As expected, interday CV values were higher than intraday values; however, lower than the recommended threshold of 5%, exhibiting satisfactory interday precision (Epshtein, 2020).

**Table 3:** Limits of detection (LLOD), limits of quantification (LLOQ), precision (intra- and interday) and carry-over of the analytical methodology for tryptophan (TRP) and serotonin (5-HT).

Item	TRP	5-HT
LLOQ, nM	2.58	0.78
LLOD, nM	8.60	2.60
Precision intraday, %^1^	2.20	2.36
Precision interday, %^1^	4.43	4.96
Carry-over, nM^2^	0.79	0.35

1 CV is the coefficient of variation;

2 residual analyte from a preceding standard analysis with 5,000 nM.

The carry-over corresponding to residual analyte from the preceding analysis of the 5,000 nM was 0.79 and 0.35 nM for TRP and 5-HT, respectively (Table 3). These values corresponded to 9.17% and 13.3% of the LLOD for the TRP and 5-HT, respectively. According to ICH guidelines M10 (ICH, 2022), carry-over in blank samples analyzed after the highest calibration standard should not exceed 20% of the analyte response at the LLOQ. Therefore, the observed carry-over had no significant effect on the analysis, representing less than 0.02% of the preceding injection.

**Fig. 2:**
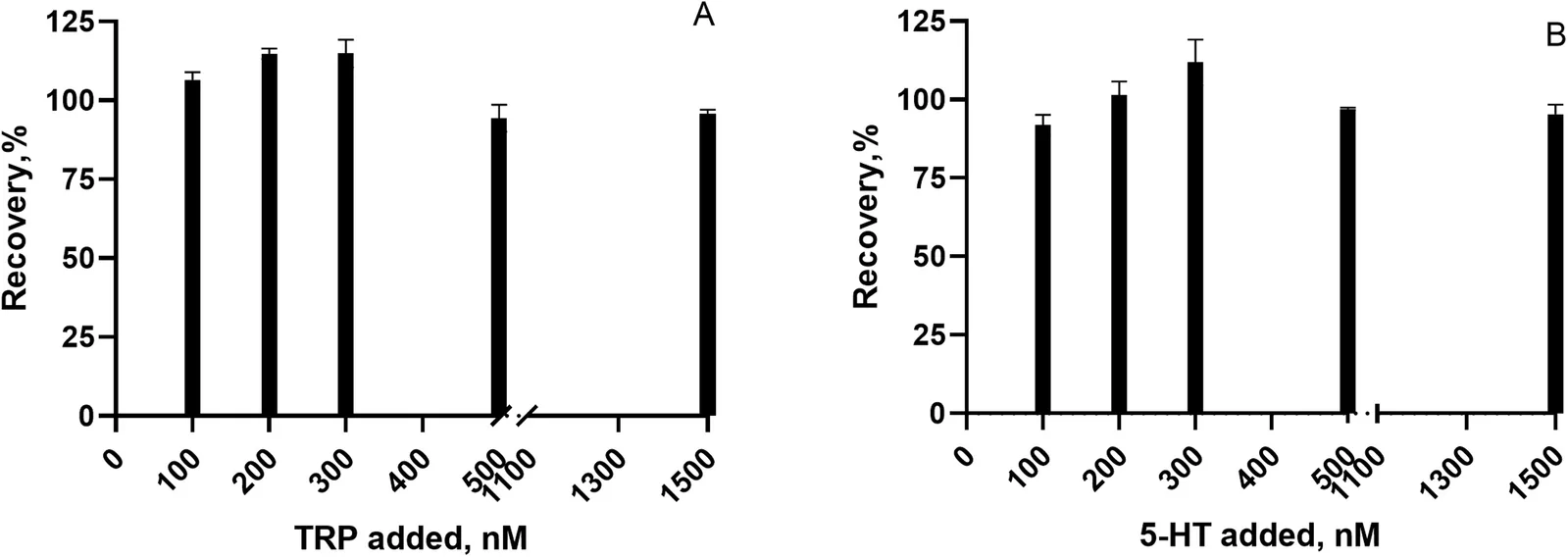
Accuracy of tryptophan (TRP; A) and serotonin (5-HT; B) quantification using standards spiked into bovine serum.

The mean accuracy was 105.2% for TRP, with accuracies of 106.5%, 114.7%, 114.8%, 94.3%, and 95.8% at spiked concentrations of 100, 200, 300, 500, and 1500 nM, respectively. For 5-HT, the mean accuracy was 99.4%, with accuracies of 91.8%, 101.5%, 111.8%, 97.0%, and 95.4% at the same spiked concentrations, respectively (Fig. 2). The recoveries of standards spiked into serum samples, which were close to 100%, confirm low matrix interference and demonstrate high accuracy for the determination of these compounds. According to ICH (2022) recommendation for sample analysis, acceptable accuracy should be within ±15% of the nominal concentration.

**Fig. 3:**
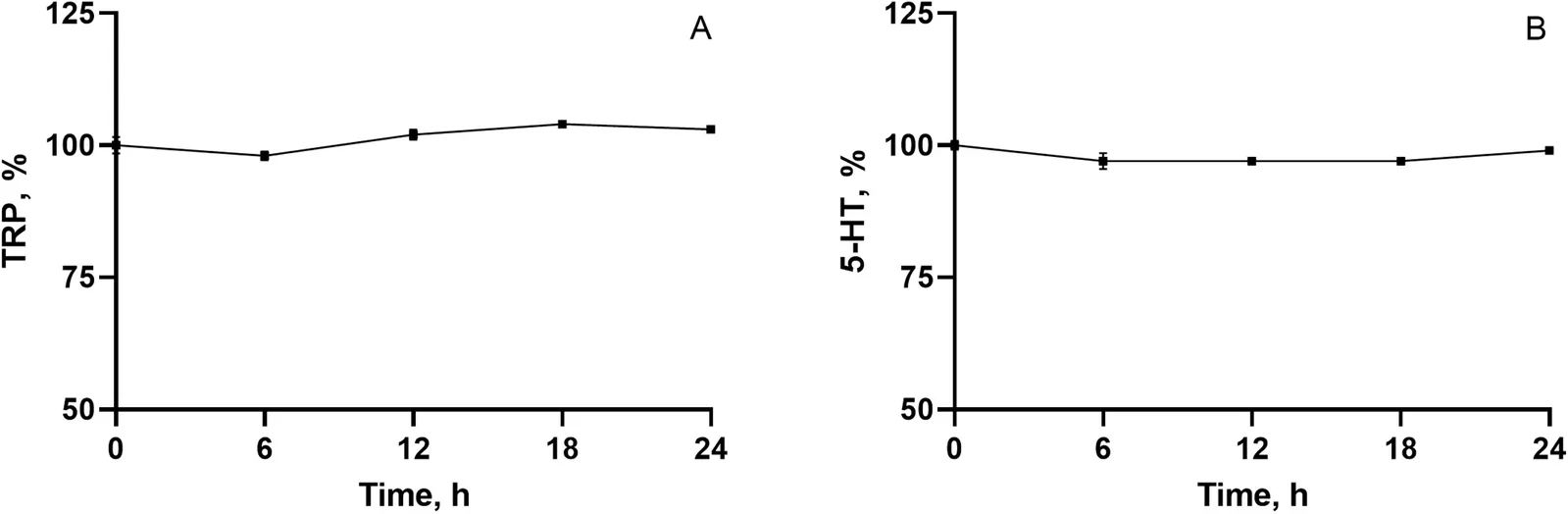
Short-term stability of serum samples maintained at room temperature (23.5 ± 0.68°C) during HPLC analysis.

Samples had a relatively constant concentration during short-term stability evaluation, with concentrations after 24 h in the HPLC at room temperature corresponding to 103% and 98% of the original concentrations for TRP and 5-HT, respectively (Fig. 3). Conversely, after 3 freeze-thaw cycles, the TRP reduced to 93 ± 1.1% and 5-HT to 95 ± 0.6% from the original concentration (baseline), respectively. When evaluating cerebrospinal fluid, (Hubbard et al., 2010) found that 5-HT remained stable in samples for at least 8 h; however, its concentration decreased markedly after three freeze–thaw cycles. The instability of TRP and 5-HT indicates that freeze–thaw cycles are not recommended for the determination of these compounds.

### Storage conditions and stability of blood samples

**Fig. 4:**
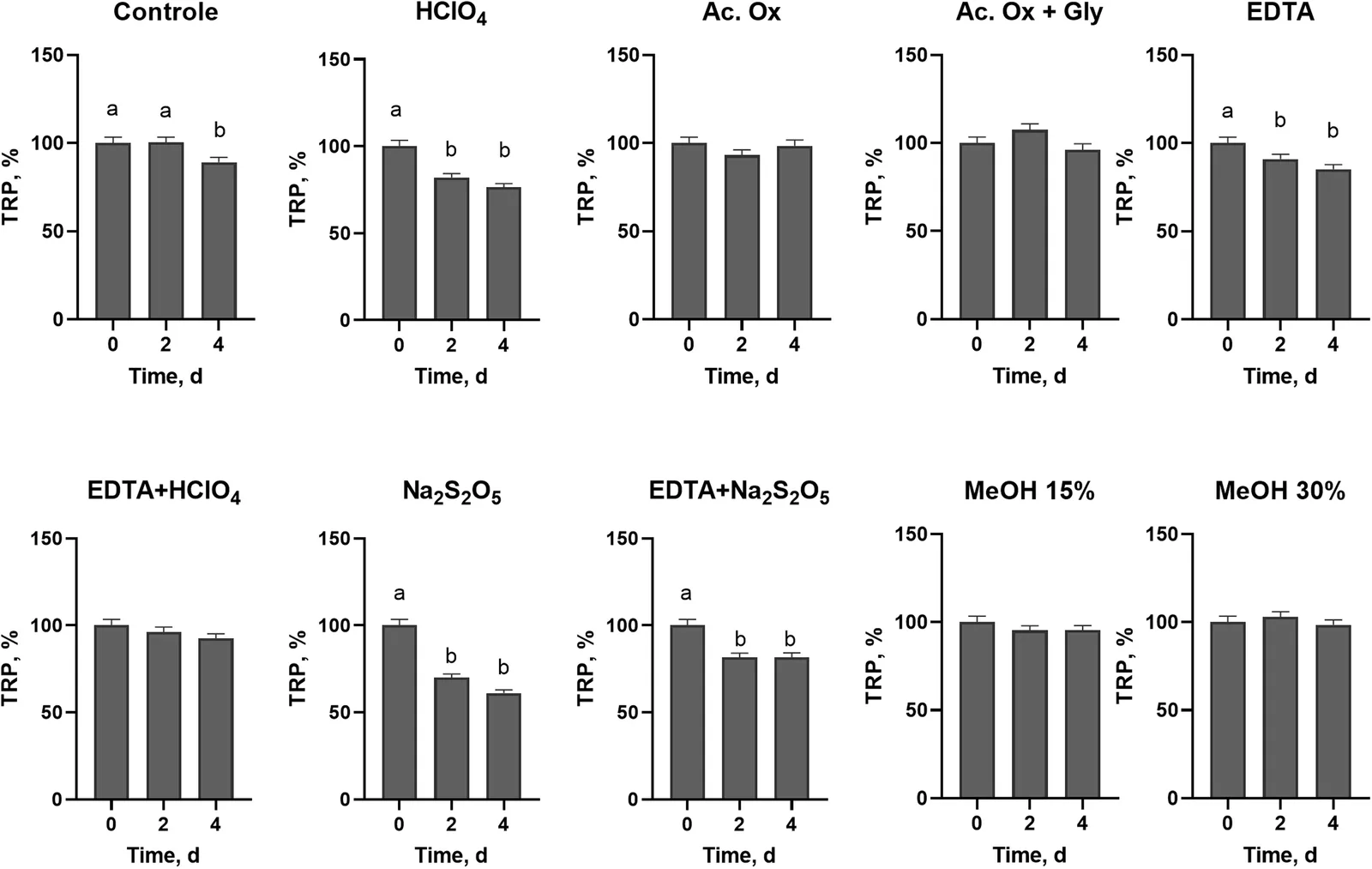
Relative concentration of tryptophan (TRP) in bovine serum stored up to 4 days at 4°C with different stabilizers. Values are expressed as mean ± standard error (n = 6). Control – no stabilizers; HClO₄ - perchloric acid (0.67 M); Ac. Ox = oxalic acid (0,01 M); Ac. Ox.+ Gly = oxalic acid (0.01 M) plus glycine (0.17 M); EDTA - ethylenediaminetetraacetic acid (0,17); EDTA+ HClO₄ - ethylenediaminetetraacetic acid (0.17) plus perchloric acid (0.67 M); Na₂S₂O₅ - sodium metabisulfite (0.08 M); EDTA + Na₂S₂O - ethylenediaminetetraacetic acid (0.17) plus sodium metabisulfite (0.08 M); MeOH 15% - methanol (15% v/v); MeOH 30% - methanol (30% v/v). Baseline concentrations were normalized to 100%.

The stability of TRP in refrigerated bovine serum (4°C) for up to 4 days was significantly affected by the preservatives used (Fig. 4). TRP degradation was observed in the control, HClO₄, EDTA, Na₂S₂O₅, and EDTA + Na₂S₂O₅ groups, with statistically significant reductions starting from the second day. In contrast, treatments with oxalic acid, oxalic acid + glycine, EDTA + HClO₄, MeOH 15% and MeOH 30% prevented TRP degradation. Stability evaluated at 4°C provides an indication of analyte losses during sample preparation and represents a practical approach for pre-selecting suitable preservatives for long-term storage.

TRP and 5-HT are sensitive to biochemical and chemical oxidative reactions (Szeitz and Bandiera, 2018; Unger et al., 2020). Among the tested conditions, only EDTA + HClO₄ and 30% methanol were effective in preventing degradation of TRP during 4 days of storage at 4°C. EDTA is an efficient metal-chelating agent and is widely recognized for its antioxidant properties, protecting analytes against oxidation during storage (Thbayh et al., 2023).

**Fig. 5:**
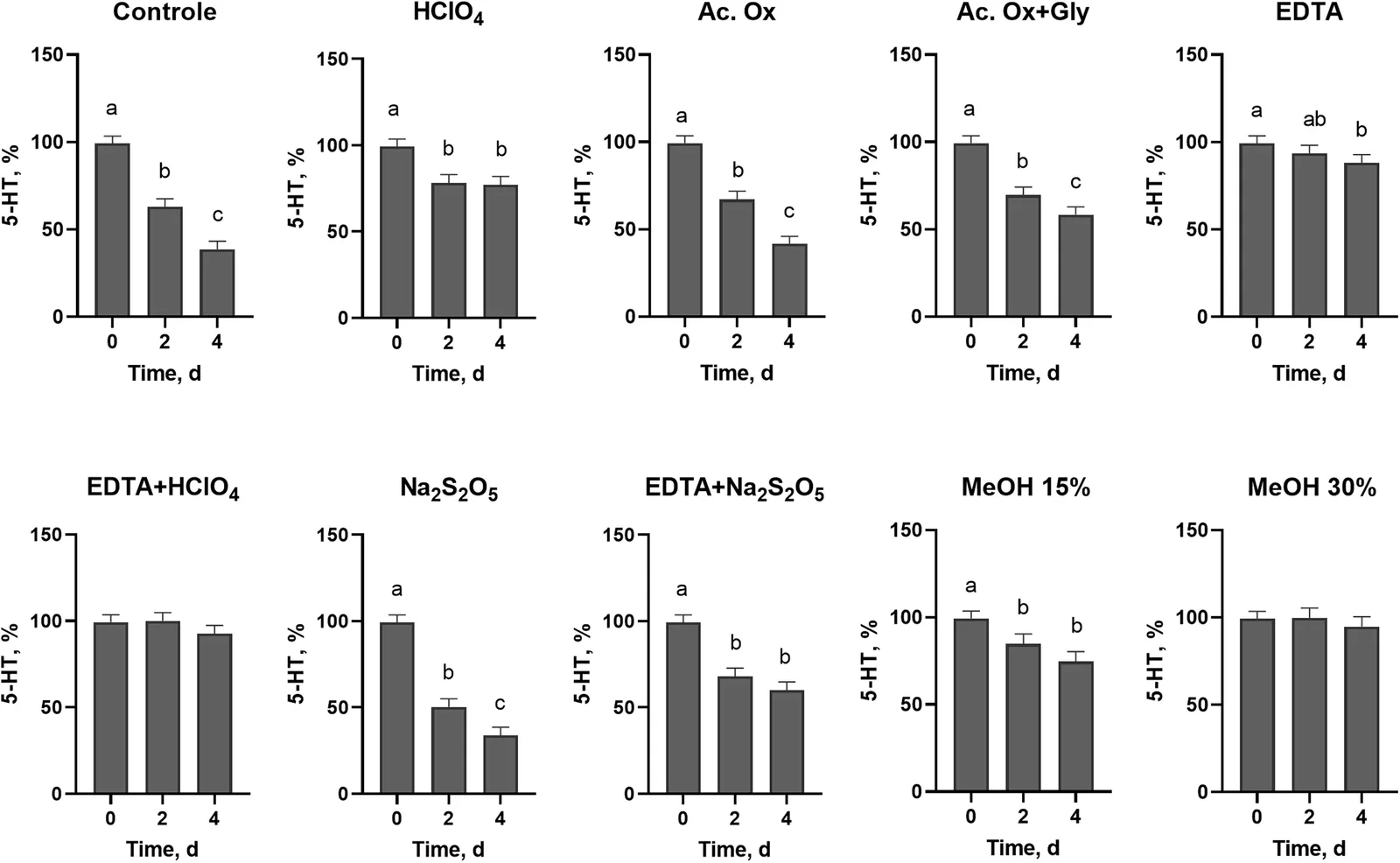
Relative concentration of serotonin (5-HT) in bovine serum stored up to 4 days at 4°C with different stabilizers. Values are expressed as mean ± standard error (n = 6). Control – no stabilizers; HClO₄ - perchloric acid (0.67 M); Ac. Ox = oxalic acid (0,01 M); Ac. Ox.+ Gly = oxalic acid (0.01 M) plus glycine (0.17 M); EDTA - ethylenediaminetetraacetic acid (0,17); EDTA+ HClO₄ - ethylenediaminetetraacetic acid (0.17) plus perchloric acid (0.67 M); Na₂S₂O₅ - sodium metabisulfite (0.08 M); EDTA + Na₂S₂O - ethylenediaminetetraacetic acid (0.17) plus sodium metabisulfite (0.08 M); MeOH 15% - methanol (15% v/v); MeOH 30% - methanol (30% v/v). Baseline concentrations were normalized to 100%.

The concentration of 5-HT in serum decreased drastically during storage at 4°C, reaching less than 50% of the original concentration after four days in control (Fig. 5). Although HClO₄ and EDTA, used individually, attenuated 5-HT loss, only their combination was effective in preventing it during short-term storage. Additionally, adding methanol at a concentration of 30% effectively maintained 5-HT concentration.

Strong acids may exert both beneficial and detrimental effects on sample stability. Sample deproteinization is an essential step in HPLC analysis, and HClO₄ is commonly used for this purpose (Piechocka and Głowacki, 2023). However, previous studies have reported that HClO₄ alone may promote 5-HT degradation (Pussard et al., 1996). In contrast, HClO₄ promotes protein denaturation and enzyme inactivation (Patel et al., 2005). Earlier investigations indicated that acidification alone is insufficient to effectively prevent 5-HT degradation (Thorré et al., 1997). In the present study, although EDTA and HClO₄ individually were not effective in preventing analyte degradation, their combined use successfully minimized losses. Likely the result of a synergistic mechanism involving metal ion chelation, which limits oxidation, together with enzyme inactivation. Methanol was also effective in improving the stability of TRP and 5-HT during storage. Its ability to denature proteins and precipitate oxidative enzymes without the harsh acidic conditions may enhance the preservation of TRP and its metabolites (Jäger et al., 2016).

**Fig. 6:**
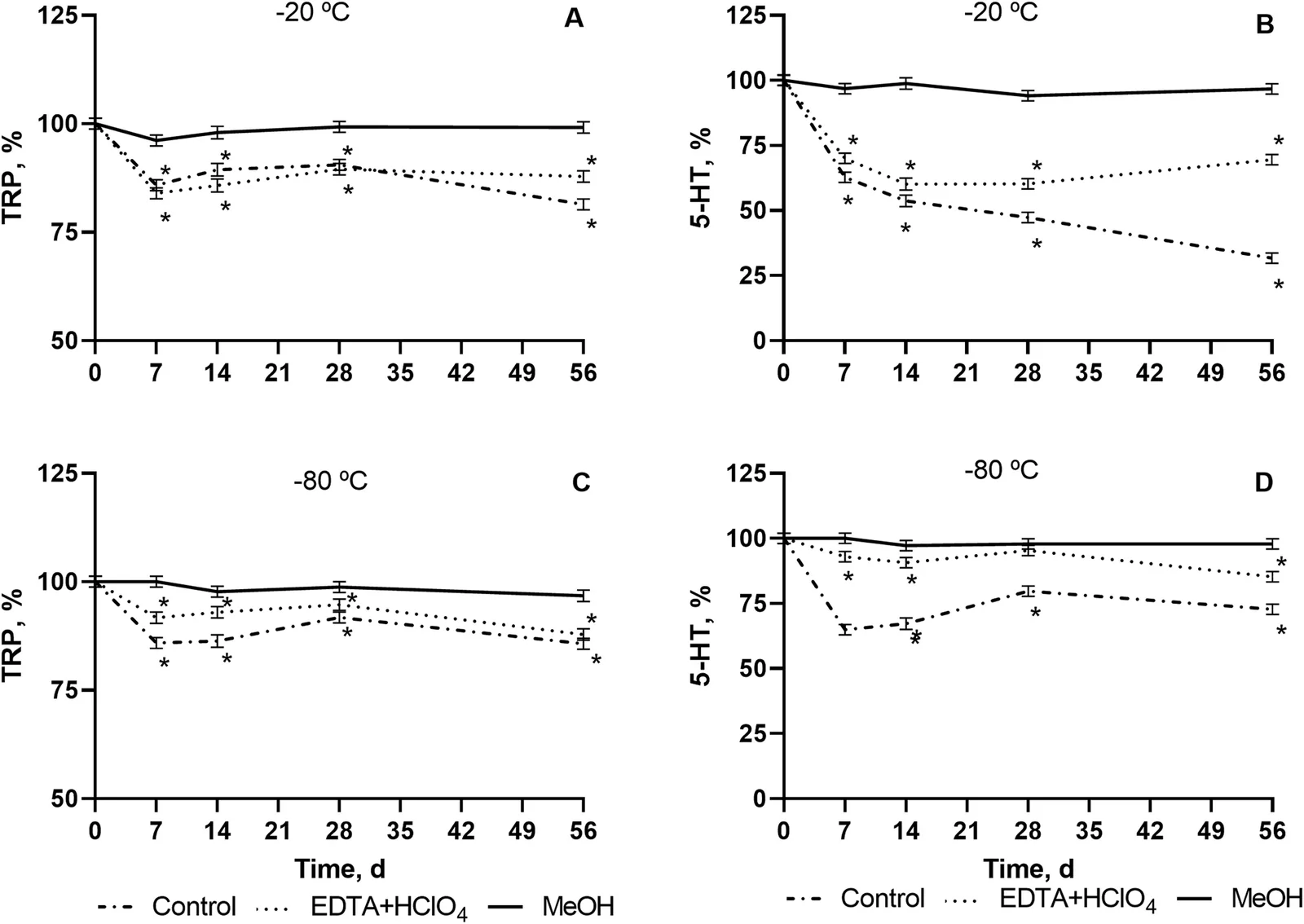
Stability of tryptophan (TRP) and serotonin (5-HT) in bovine serum without stabilizer (Control), addition of perchloric acid plus EDTA (EDTA+HClO₄), or with methanol (30% v/v; MeOH), stored at -20 °C and -80°C for up to 56 days. *Indicates a significant difference relative to the original concentration. Baseline concentrations were normalized to 100%.

Storage of the samples at −20°C and −80°C didn´t prevent degradation of TRP and 5-HT, with a reduction in their original concentrations as early as 7 days in control samples (Fig. 6). However, the degradation was faster at -20 °C than at -80 °C. Although the addition of EDTA + HClO₄ reduced 5-HT degradation, these compounds could not prevent significant changes in the concentrations of 5-HT and TRP in the samples. The addition of methanol effectively maintained 5-HT and TRP concentrations in the samples for 56 days, regardless of freezing temperature.

The freezing temperature, particularly −80°C, slowed sample degradation. However, freezing does not completely prevent the activity of degradative enzymes or oxidation reactions, which may lead to a gradual loss of sample integrity over time. Although the addition of EDTA + HClO₄ reduced losses of 5-HT, this combination was not sufficient to prevent significant changes in TRP and 5-HT concentrations during long-term storage. In contrast, the addition of methanol effectively maintained TRP and 5-HT concentrations for 56 days, regardless of freezing temperature. Enzymatic inhibition induced by methanol has been reported as one of its main stabilizing mechanisms when used in biological samples (Carré et al., 2025).

### 5-HT and TRP concentration in blood fractions

**Fig. 7:**
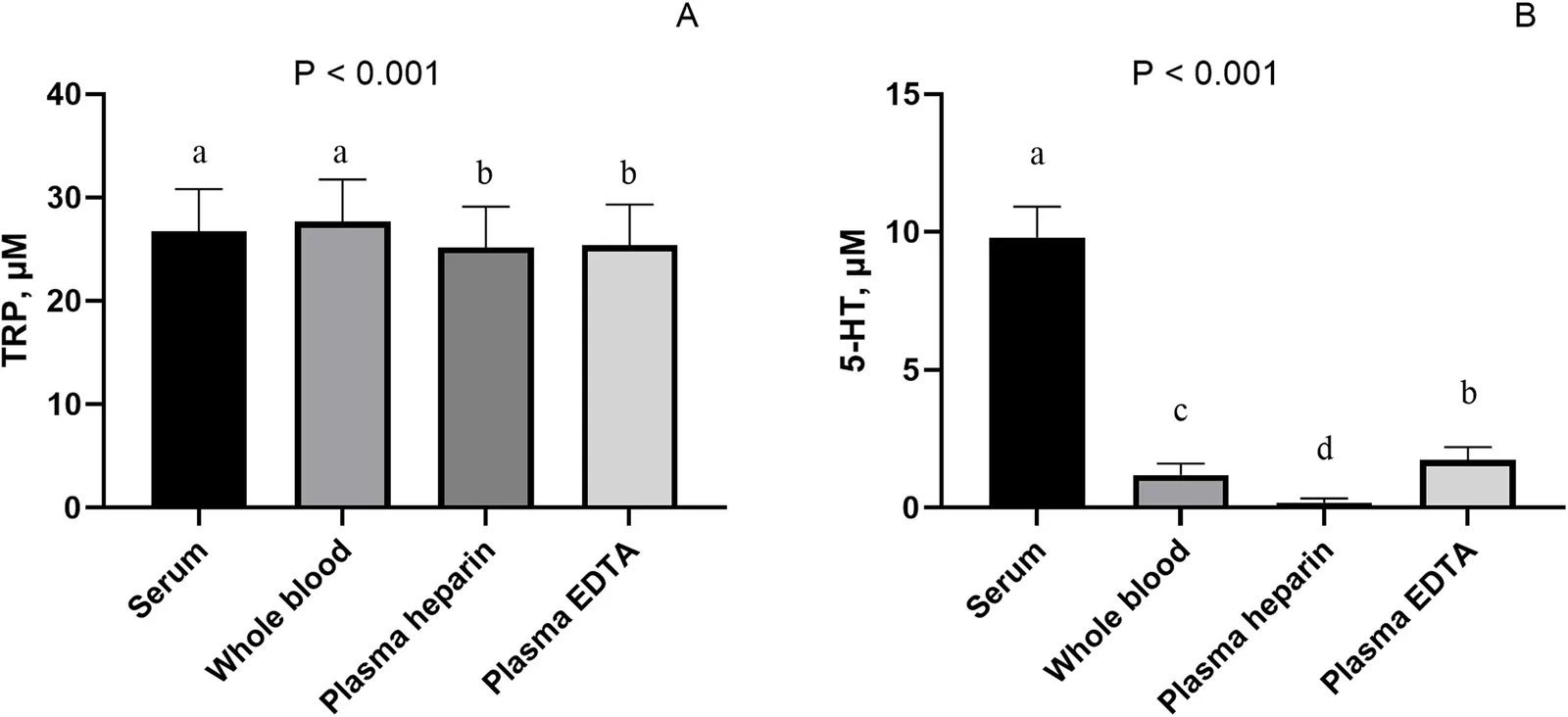
Concentration of (A) tryptophan (TRP) and (B) serotonin (5-HT) in serum, whole blood, plasma heparin, and plasma EDTA.

TRP concentrations showed slight differences among blood fractions. Serum and whole blood exhibited concentrations approximately 7% higher (P < 0.05) than plasma obtained with heparin or EDTA (Fig. 7). Although the difference in TRP concentrations between serum and plasma is small, it is consistent and should be considered when comparing analytical results. The matrix effects on tryptophan concentration observed in this study corroborate with previous findings (Metri et al., 2023). Clot-related biochemical changes and the release of intracellular components from platelets are likely responsible for the observed differences in TRP concentrations between serum and plasma (Lee et al., 2017).

The concentration of 5-HT showed marked variation among blood fractions, with differences (P < 0.05) observed between all of them. The highest concentration was found in serum (9.51 µM), whereas values in the other fractions were considerably lower: 1.73 µM in EDTA plasma, 0.19 µM in heparinized plasma, and 1.19 µM in whole blood. Besides influencing analytical results, the choice of blood fraction also has biological implications. Most of the circulating 5-HT in the body is present in platelets and very little 5-HT in plasma (Szeitz and Bandiera, 2018). During clot formation, 5-HT is released (Cerrito et al., 1993). Thus, plasma serotonin reflects the concentration of free 5-HT in circulation, whereas serum serotonin represents the total circulating pool (both the free circulating fraction and the serotonin released from platelets during coagulation (Mercado and Kilic, 2010).

Serum 5-HT concentrations were markedly higher than those observed in the other fractions, likely reflecting total circulating 5-HT. In contrast, whole blood, heparinized plasma, and EDTA plasma showed closer concentrations, though still significantly distinct, suggesting that platelets may have partially released 5-HT in these fractions. The present results are consistent with previous studies showing that plasma 5-HT concentrations are several-fold lower than those in serum and whole blood across different species (Szeitz and Bandiera, 2018; Valente et al., 2021).

The difference between heparin and EDTA in biochemical analyses has been previously reported (Mohri et al., 2007). However, to the best of our knowledge, differences in 5-HT concentrations between heparin and EDTA plasma have not been evaluated previously in bovines. The intermediate concentration of 5-HT observed in whole blood, relative to plasma and serum, suggests that acid deproteinization partially releases platelet-stored 5-HT, potentially compromising repeatability and analytical consistency. The differences in 5-HT concentrations between plasma samples obtained with different anticoagulants may be related to the distinct mechanisms by which EDTA and heparin prevent coagulation (Lima-Oliveira et al., 2021), which may differentially affect platelet activation and subsequent 5-HT release. Analytical interference from the anticoagulants is unlikely, given the chromatographic separation and fluorescence detection employed in the present method, which provides high selectivity for naturally fluorescent compounds such as 5-HT. A low correlation between serum and EDTA plasma 5-HT concentrations has also been reported in cats (Ramos et al., 2021), supporting the potential influence of blood fraction on 5-HT measurements. Overall, the substantial differences observed among blood matrices highlight the importance of selecting the appropriate blood fraction when interpreting and comparing 5-HT concentrations across studies.

## Conclusion

The proposed HPLC-FLD method is sensitive and reliable for the simultaneous quantification of TRP and 5-HT in blood samples. Although both analytes exhibit instability even under frozen storage, the addition of 30% methanol effectively preserves TRP and 5-HT at −20°C and −80°C for at least 56 days, ensuring sample stability during long-term storage. However, freeze–thaw cycles are not recommended for the determination of these compounds. While TRP concentrations were similar among blood fractions, 5-HT levels showed considerable variability, with higher concentrations observed in serum. Future studies should explore the relationship between 5-HT concentrations in different blood fractions and their associated biological responses.

## Statement for studies in animals

The experimental procedures were approved by the Ethics Committee on the Use of Animals of Western Parana State University under protocol 018/2025.

## Ethics declaration

This study was conducted in accordance with the following guidelines for animal welfare and/or reporting: Brasilian National Council for the Control of Animal Experimentation (CONCEA). This study was approved by the Ethics Committee on the Use of Animals of Western Parana State University. (Approval No. 018/2025).

## Funding

This study was supported by Coordenação de Aperfeiçoamento de Pessoal de Nível Superior - CAPES (Grant No. 001; graduate scholarships to R.C.M.), and Conselho Nacional de Desenvolvimento Científico e Tecnológico - CNPq (Grant No. 308889/2022-3).

## CRediT authorship contribution statement

**Ryana Cristina Markmann:** Writing – original draft, Methodology, Formal analysis, Data curation, Conceptualization. **Maximiliane Alavarse Zambom:** Writing – review & editing, Visualization. **Gilberto Costa Braga:** Writing – review & editing, Visualization. **Julio César Meyer:** Data curation. **Maria Luiza Fischer:** Data curation, Conceptualization. **Sidnei Antônio Lopes:** Writing – review & editing, Visualization. **David Lee Harmon:** Writing – review & editing, Writing – original draft. **Ériton Egidio Lisboa Valente:** Writing – review & editing, Writing – original draft, Supervision, Software, Methodology, Conceptualization.

## Declaration of competing interest

The authors declare the following financial interests/personal relationships which may be considered as potential competing interests:

Eriton Egidio Lisboa Valente reports financial support was provided by Conselho Nacional de Desenvolvimento Científico e Tecnológico. Ryana C. Markmann reports financial support was provided by Coordenação de Aperfeiçoamento de Pessoal de Nível Superior. If there are other authors, they declare that they have no known competing financial interests or personal relationships that could have appeared to influence the work reported in this paper.

## Data Availability

Data will be made available on request.
